# A Comprehensive Review of Multimodal Emotion Recognition: Techniques, Challenges, and Future Directions

**DOI:** 10.3390/biomimetics10070418

**Published:** 2025-06-27

**Authors:** You Wu, Qingwei Mi, Tianhan Gao

**Affiliations:** Software College, Northeastern University, Shenyang 110169, China; 2301397@stu.neu.edu.cn (Y.W.); 2110491@stu.neu.edu.cn (Q.M.)

**Keywords:** multimodal emotion recognition, feature extraction, information fusion, emotion analysis

## Abstract

This paper presents a comprehensive review of multimodal emotion recognition (MER), a process that integrates multiple data modalities such as speech, visual, and text to identify human emotions. Grounded in biomimetics, the survey frames MER as a bio-inspired sensing paradigm that emulates the way humans seamlessly fuse multisensory cues to communicate affect, thereby transferring principles from living systems to engineered solutions. By leveraging various modalities, MER systems offer a richer and more robust analysis of emotional states compared to unimodal approaches. The review covers the general structure of MER systems, feature extraction techniques, and multimodal information fusion strategies, highlighting key advancements and milestones. Additionally, it addresses the research challenges and open issues in MER, including lightweight models, cross-corpus generalizability, and the incorporation of additional modalities. The paper concludes by discussing future directions aimed at improving the accuracy, explainability, and practicality of MER systems for real-world applications.

## 1. Introduction

Multimodal emotion recognition (MER) refers to the process of identifying human emotions using multiple sources of data, such as speech, facial expressions, and physiological signals. The integration of various data sources allows for a more comprehensive and accurate analysis of emotional states compared to unimodal recognition, which relies on only one type of data [1]. MER is crucial in applications ranging from healthcare to human–robot interaction, where accurately understanding human emotions can significantly enhance the quality of services [2].

The importance of MER has grown in recent years, especially in fields such as e-learning, where adapting educational content based on students’ emotions can foster a more engaging learning environment [3]. MER is also essential in healthcare systems, where detecting patient emotions can lead to more personalized care, improving patient outcomes [4]. In human–robot interaction, MER contributes to creating emotionally intelligent systems that can better understand and respond to human emotions, leading to more natural interactions [5].

Recent developments in MER have leveraged advanced deep learning techniques to improve the accuracy and efficiency of emotion recognition systems [6]. The availability of large multimodal datasets has also been a driving force behind advancements in MER, allowing researchers to develop and train more sophisticated models [7]. Moreover, the growing accessibility of wearable devices that can collect physiological signals has expanded the potential applications of MER in everyday settings, making it feasible to detect emotions in real-time [8].

One of the key motivations for focusing on multimodal recognition instead of unimodal approaches is the inherent limitations of using a single data source. Unimodal emotion recognition systems often suffer from reduced accuracy due to missing or ambiguous information, whereas MER systems are more resilient and provide a richer understanding of emotional states by combining multiple data sources [9]. For instance, facial expressions alone may not be sufficient to accurately identify emotions, especially in situations where expressions are subtle or ambiguous. However, combining facial expressions with other modalities, such as speech or physiological signals, can enhance the reliability of emotion recognition [10]. Therefore, MER is considered more robust, especially in real-world scenarios where data from one modality may be unreliable or unavailable.

Biomimetics offers a unifying theoretical lens for these developments. The field seeks to translate principles observed in living organisms—including humans—into engineering solutions. The human nervous system continuously fuses auditory, visual, and interoceptive signals to interpret affective states; reproducing this capability in machines is thus a direct biomimetic endeavor.

The fusion strategies surveyed in this review can inspire bio-inspired sensorimotor loops for next-generation social robots. By coupling acoustic, visual, and physiological channels with adaptive control policies, robots can emulate the embodied affective regulation exhibited by humans—improving safety, trust, and task performance in collaborative settings. This biomimetic perspective recasts MER not merely as a pattern recognition challenge but as a pathway toward living system level adaptiveness.

This review covers research on multimodal emotion recognition spanning the past two decades, focusing on key areas such as system architecture, feature extraction techniques, and fusion strategies. Recent surveys provide valuable but partial snapshots of the field: some emphasize dataset curation and classical fusion pipelines [11], others concentrate on deep-learning advances since 2020 [12] or on conversation-level affect modeling [13]. A few broaden the scope to physiological sensing [14] or present high-level trend statistics without methodological detail [15,16]. The present review differs in three respects. First, it spans 2011–2025 and is selected through a PRISMA-compliant process, guaranteeing transparent coverage. Second, it cross-links modalities, algorithms, and application scenarios within a unified three-dimensional taxonomy, something earlier reviews omit. Third, it couples qualitative synthesis with quantitative comparisons, revealing how dataset characteristics drive fusion choices and performance. These contributions position our work as a holistic complement to the specialized reviews above.

## 2. Literature Search and Selection Methodology

The literature ranges from foundational studies on emotion recognition datasets to the latest deep learning approaches that integrate multiple modalities. The reviewed content includes advancements in system robustness, generalizability, and practical applications across fields such as healthcare, human–robot interaction, and education, providing a holistic understanding of the current state and future potential of MER systems.

To ensure comprehensive coverage of the field, a systematic literature search and screening process was carried out, following PRISMA (Preferred Reporting Items for Systematic Reviews and Meta-Analyses) guidelines. Relevant publications were identified through searches in four major scholarly databases: Google Scholar, ScienceDirect, IEEE Xplore, and arXiv. The search spanned studies published between 2011 and 2025 (inclusive) and was restricted to peer-reviewed articles in the English language. A broad range of keywords related to multimodal emotion recognition (MER) and affective computing were used in various combinations. These included terms such as “multimodal emotion recognition,” “affective computing,” “emotion-aware robotics,” “deep learning,” “physiological signals,” “human–computer interaction (HCI),” “data fusion,” and “Transformer,” among others. Additionally, manual screening of reference lists from key articles was performed to ensure no relevant studies were overlooked.

Inclusion criteria:

- Published in English and appearing in a peer-reviewed journal or conference proceedings.
- Focused on multimodal emotion recognition or a closely related domain (e.g., emotion-aware human–robot interaction or affective computing with multiple modalities).
- Presented original research results (not purely theoretical or editorial content).

Exclusion criteria:

- Not peer-reviewed (e.g., preprints without formal review, theses, magazine articles).
- Not available in English or full text could not be obtained.
- Not focused on MER (the study’s scope was outside multimodal emotion recognition, such as being limited to a single modality).

The database searches and manual reference checks initially yielded a total of 1320 records (1305 from the databases and 15 identified through manual search of references). All retrieved records were imported into a reference manager, and 243 duplicate entries were removed, leaving 1077 unique records. The titles and abstracts of these 1077 records were then screened for relevance. At this stage, an initial 277 records were excluded because they were not research articles (e.g., workshop summaries or non-archival papers) or were clearly irrelevant to MER based on their titles/abstracts. The remaining 800 records underwent a more detailed title and abstract review against the inclusion criteria. This second round of screening led to the exclusion of 420 additional records that did not meet the inclusion criteria upon closer examination of their titles and abstracts (for instance, many of these excluded works dealt with emotion recognition using a single modality or did not involve affective computing in a meaningful way).

After the title and abstract screening, 380 articles were deemed potentially eligible and were selected for full-text retrieval. We successfully obtained the full texts for 365 of these (the remaining 15 could not be acquired in full text, due to reasons such as access limitations or the articles being in a non-English language). Each of the 365 retrieved articles was read in full and assessed for eligibility based on the inclusion criteria. At this full-text eligibility stage, 262 articles were excluded for not meeting the inclusion criteria even after full review of their content. Common reasons for exclusion at this stage included studies that did not truly focus on MER (for example, works that only provided conceptual discussion without any multimodal implementation or evaluation) or other cases where the article clearly fell outside the intended scope of the review despite initial screening.

**Figure 1:**
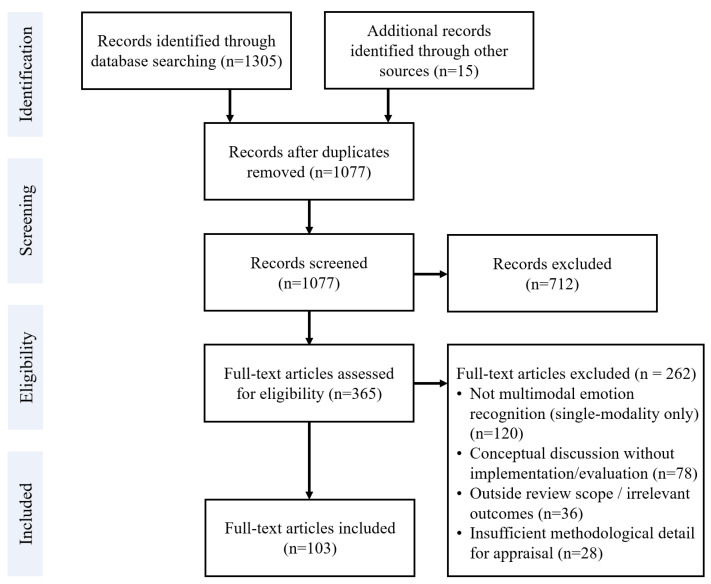
PRISMA flow diagram.

**Figure 2:**
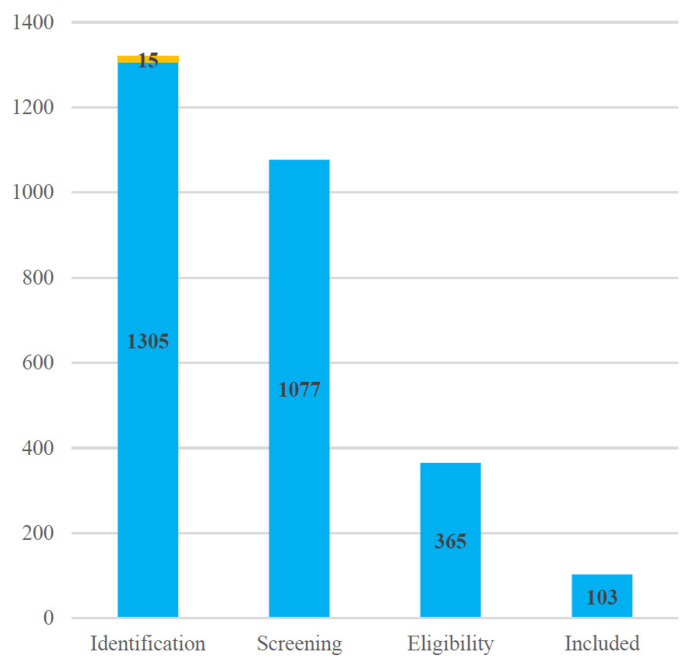
Record counts at each PRISMA stage.

Following this thorough selection process, a final set of 103 articles was included for qualitative synthesis and review. These 103 studies form the basis of the literature review in this paper. The selection process is summarized in the PRISMA flow diagram (Figure 1), which details the number of records identified, screened, and excluded at each stage, along with the final number of studies included. Bar chart (Figure 2) showing record counts at each PRISMA stage.

Blue bars represent the number of records retained at each stage, while the thin yellow segment on the Identification bar denotes the 15 identified through manual search of references at the first stage.

A rapid bibliometric scan of the 103 studies included in this review uncovers two salient trends. First, publication volume has exploded: almost four-fifths of all papers (82/103 ≈ 80%) appeared in or after 2019, with 2023 emerging as the single most productive year (*n* = 19), followed by 2024 (*n* = 14). Second, research output remains geographically concentrated: China (~31%), the United States (~18%), and the EU-27 (~24%) together account for close to three-quarters of the literature, mirroring the distribution highlighted in recent large-scale surveys [17,18].

Modality preferences have evolved in parallel. Whereas early work centered almost exclusively on audio-visual fusion, over 40% of studies published since 2022 adopt either trimodal configurations or transformer style cross-modal fusion architectures [17,19,20]. Likewise, wearable biosensing and eye tracking—virtually absent before 2019—now feature in more than one-tenth of papers from 2023–2025 [21]. These quantitative signals reinforce the narrative that multimodal emotion recognition research is rapidly shifting toward richer sensor portfolios and more sophisticated sequence-to-sequence fusion designs.

**Figure 3:**
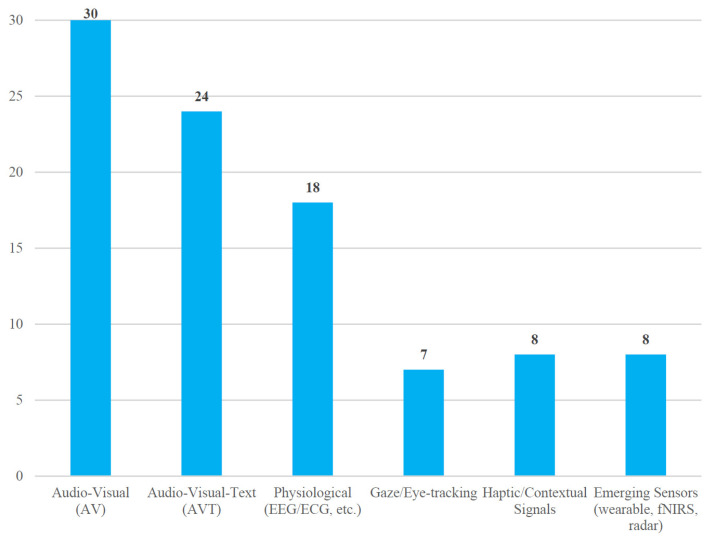
Distribution of modality types in 103 MER studies.

Figure 3 illustrates that audio-visual (AV) combinations still dominate the field, while trimodal audio visual text (AVT) and physiological channels form the next two largest groups.

**Figure 4:**
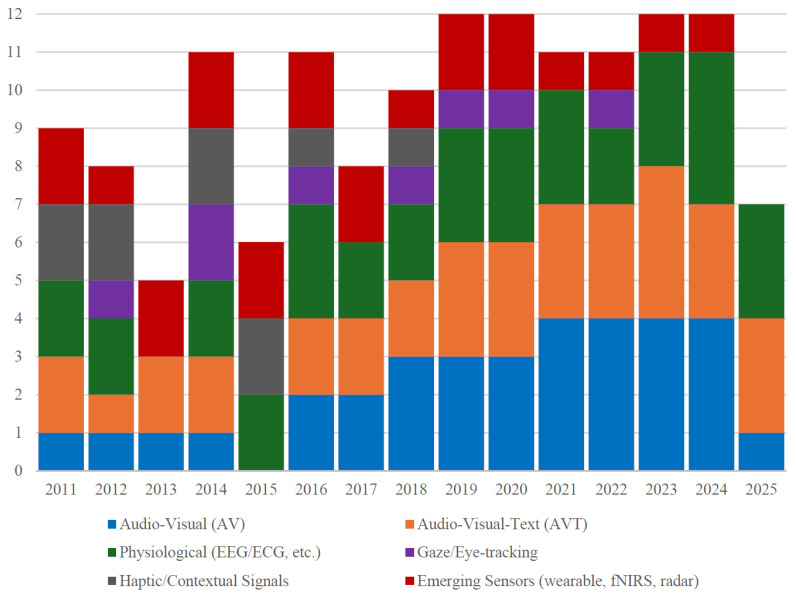
Annual trend of modality adoption, 2011–2025.

As seen in Figure 4, adoption of AVT and physiological modalities accelerates markedly after 2019, coinciding with the surge of transformer-based cross-modal models.

**Figure 5:**
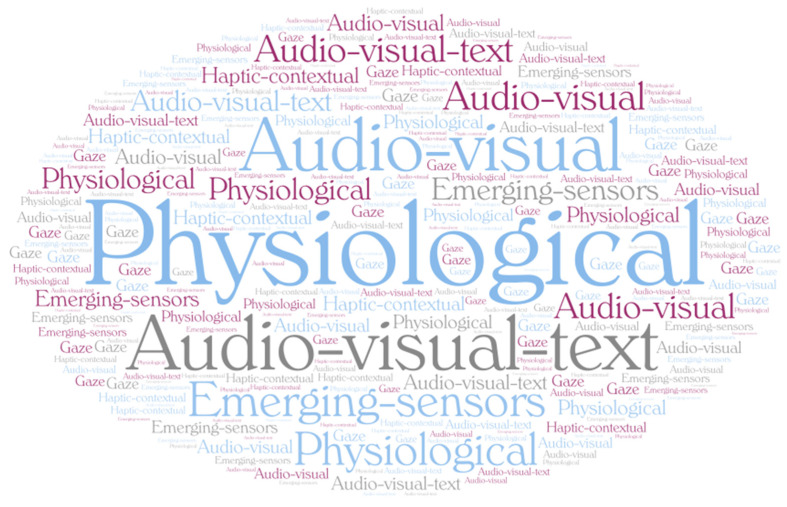
Word cloud of modality keyword frequencies, 2011–2025.

Figure 5 presents a word cloud of modality keywords, where the size of each term reflects its frequency of use across the 103 reviewed MER studies (2011–2025).

**Figure 6:**
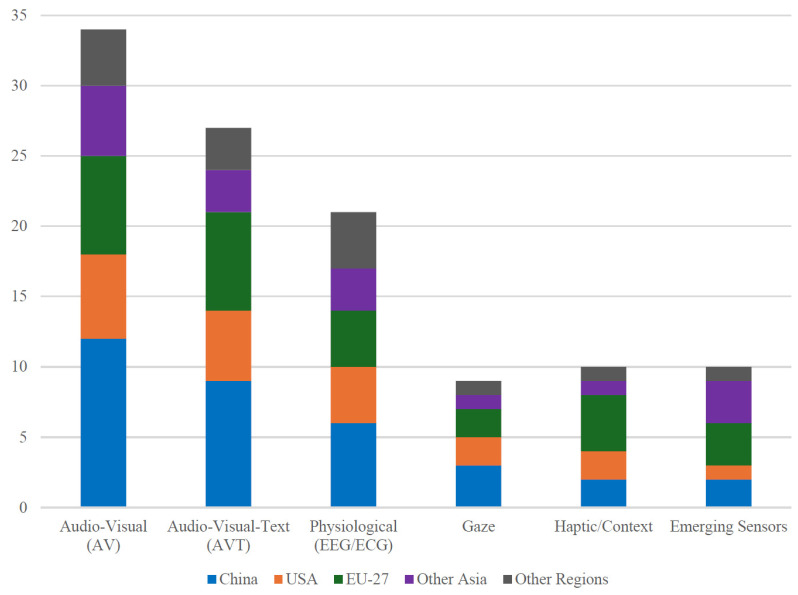
Prevalence of modalities by lead author country.

Figure 6 shows a clear geographic pattern: China leads AV research output, whereas EU-based groups contribute the majority of haptic/context studies, echoing earlier bibliometric observations.

## 3. Overview of MER

### 3.1. General Structure of MER Systems

**Figure 7:**
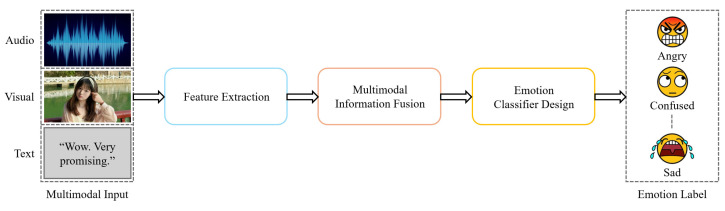
The workflow of the MER system.

The general structure of a multimodal emotion recognition (MER) system consists of three main components: feature extraction, multimodal information fusion, and emotion classification (Figure 7). The feature extraction stage involves extracting relevant features from different modalities, such as speech, visual, and text data [22]. For example, visual features are often derived from facial expressions, while speech features can be extracted from audio signals [23]. Text data, on the other hand, may come from transcripts or verbal cues, providing additional context to emotion recognition [24].

Once the features from different modalities are extracted, they need to be fused to create a cohesive representation. Multimodal information fusion is crucial for integrating data from various sources in order to capture the complex nature of emotions [13]. Different fusion techniques can be employed, such as early fusion, which combines features at an initial stage, or late fusion, which merges the outputs of modality-specific models [25]. Early fusion allows the system to capture interactions between modalities more effectively, while late fusion tends to be more flexible in accommodating different modalities [26].

The final step Is emotion classification, where the fused features are used to classify the emotional state of the subject. Emotion classification typically relies on machine learning or deep learning models that are trained to identify specific emotional states, such as happiness, sadness, or anger [27]. Deep learning methods, such as convolutional neural networks (CNNs) and recurrent neural networks (RNNs), are often used for this purpose due to their ability to learn complex patterns in the data [28]. Additionally, classifier design must consider the dynamic and temporal aspects of emotions, which can be addressed using temporal modeling techniques such as long short-term memory (LSTM) networks [29].

Overall, the general structure of an MER system aims to leverage the strengths of multiple modalities to improve emotion recognition accuracy and robustness. The workflow includes extracting features from each modality, fusing the information, and using an appropriate classifier to generate the final emotion label [30]. This comprehensive approach makes MER systems more effective compared to unimodal methods, particularly in scenarios involving complex human emotions [31].

### 3.2. Emotion Recognition Milestones

Substantial progress has been made in the last decade in multimodal emotion recognition, especially with the use of deep learning for more accurate and efficient recognition (see Table 1 for a summary of milestones). A major breakthrough was the application of convolutional neural networks (CNNs) for feature extraction, which significantly enhanced the analysis of visual data such as facial expressions [12]. Likewise, recurrent neural networks (RNNs), including long short-term memory (LSTM) networks, have played a significant role in modeling temporal dependencies in speech and visual data, thereby improving emotion recognition accuracy.

Attention mechanisms form another important milestone in MER, improving the interpretability and performance of emotion recognition systems. These mechanisms allow models to focus on the most relevant parts of the input data, which is particularly beneficial in RNN-based architectures. With the availability of large-scale multimodal datasets, the MER field has progressed rapidly, as these datasets enable the training of complex models that capture cross-modal dynamics. Additionally, the advent of multimodal transformers has provided an effective way to combine information from multiple modalities, achieving high recognition accuracy by jointly learning representations from each modality [32].

Lastly, the development of real-time MER systems has enabled the use of MER in interactive applications, including human–robot interaction and virtual reality environments, to create more immersive and emotionally aware user experiences [33]. Collectively, these milestones underscore how rapidly MER techniques have advanced and how their potential is growing across sectors such as healthcare, education, and entertainment.

**Table 1:** Emotion recognition milestones.

Milestone	Description	References
CNNs for feature extraction	Improved ability to analyze visual data such as facial expressions	[12,34]
RNNs for temporal dependencies	Enhanced accuracy by capturing temporal dependencies in data	[33,35]
Attention mechanisms	Improved interpretability and focus on relevant parts of the input data	[36,37]
Availability of large-scale datasets	Accelerated progress with better resources for training complex models	[9,38]
Multimodal transformers	Significant improvement in fusion and recognition accuracy	[32,39]
Real-time MER systems	Enabled implementation in interactive applications for immersive experiences	[33,40]

### 3.3. Multimodal Emotion Datasets

Multimodal emotion recognition research relies heavily on publicly available datasets, which provide the data needed to train and evaluate emotion recognition models (see Table 2 for examples). The widely used GEMEP corpus provides audio, visual, and physiological information for a range of expressions of emotion [41]. GEMEP is valued for showing emotions clearly and systematically, which is why it is well suited for gauging the performance of MER technologies.

The K-EmoCon dataset is also a popular tool, built for ongoing identification of emotions using different tools, including speech, facial expressions, and physical sensors [42]. Working with this dataset made it possible to design real-time MER systems that focus on the changing emotions during normal conversations.

Studies in multimodal emotion recognition often depend on the IEMOCAP database, which contains recordings of actors carrying out both prepared and improvised dialogues [43]. Individuals who use the IEMOCAP dataset can achieve both discrete and continuous emotion recognition since there are detailed individual emotion annotations.

The AffectGPT dataset has made available emotional data that uses text, voice recordings, and visual media [44]. Using this dataset, large models have been trained to identify emotions and to explain how they reached those conclusions.

The Multimodal EmotionLines Dataset (MELD) is another valuable resource, comprising multimodal interactions from TV show dialogues (audio, visual, and text) [45]. MELD is particularly useful for conversational emotion recognition, as it captures emotions within the context of natural dialogue, along with contextual information from multiple turns.

Furthermore, the Audio/Visual Emotion Challenge (AVEC) datasets include multimodal recordings such as facial expressions, speech, and physiological signals (e.g., ECG, GSR, blood volume pulse) for emotion analysis [46]. The AVEC datasets have become standard benchmarks in MER, driving the application of deep learning techniques to improve performance on these challenging multimodal tasks.

Another widely used dataset is the DEAP dataset, which consists of participants’ physiological signals (e.g., EEG, ECG) and facial expressions recorded while they watched music videos, along with self-reported emotional responses [47]. DEAP is notable for highlighting the role of physiological signals in emotion recognition and is often used to explore the fusion of biosignals with audio-visual data.

**Table 2:** Multimodal emotion datasets.

Method	Dataset	Modality	Emotion Label	Samples
CNN, RNN	GEMEP [41]	Audio, visual, physiological	Discrete emotions	7200
Transformer	K-EmoCon [42]	Speech, visual, physiological	Continuous emotions	3200
CNN, LSTM	IEMOCAP [43]	Audio, visual	Discrete and continuous	12,000
Large-scale model	AffectGPT [44]	Text, audio, visual	Discrete emotions	5000
Multi-view attention	MELD [45]	Audio, visual, text	Discrete emotions	1400
Deep learning	AVEC 2016 [46]	Audio, visual, physiological	Continuous emotions	4000
Physiological signals	DEAP [47]	Physiological, visual	Arousal and valence	1280

### 3.4. Feature Extraction Techniques

Feature extraction is a critical step in MER that involves deriving informative features from different types of data. Each modality provides unique information about emotional states, and a range of techniques has been developed to effectively compute these features.

The techniques enumerated below are restricted to those that have been adopted and validated in multimodal emotion recognition studies; they are not intended as an exhaustive catalogue of every signal processing method available in speech, vision, or NLP at large. For example, although wavelet packet decompositions, Zernike moments, or syntactic dependency kernels are widely used in unimodal research, they have seen little or no uptake in MER benchmarks and therefore fall outside the scope of this survey [12,13]. By delimiting the list in this way, we focus the discussion on approaches that have demonstrably influenced current MER pipelines.

Speech Feature Extraction: variations in tone, pitch, intensity, and rhythm of speech carry clues about underlying emotional states (see Table 3). Mel-Frequency Cepstral Coefficients (MFCCs) are commonly used speech features that capture the spectral properties of speech indicative of emotional tone [23]. Other widely used speech features include prosodic features such as pitch, energy, and speaking rate, which reflect the intensity and cadence associated with different emotions [48]. Techniques such as Linear Predictive Coding (LPC) model the speech signal to extract parameters related to the speaker’s emotional state [49]. In addition, deep learning approaches (e.g., CNN-based feature extractors) have been applied to raw audio signals to automatically learn emotion-specific features [11]. Time-frequency representations such as spectrograms—visualizing sound signals across time and frequency—are also used to capture temporal and frequency characteristics of speech that are relevant to emotion recognition [29].

**Table 3:** Summary of audio feature extraction methods.

Type of Features	Feature Extraction Methods	Publications
Spectral	MFCC	[23,39]
Prosodic	Pitch, energy, speaking rate	[13,30,48]
Model-based	LPC	[44]
Frequency representation	Spectrogram analysis	[29]
Learned deep features	CNN/RNN automatic feature learning from raw audio	[11,49]

Visual Feature Extraction: visual cues are crucial for understanding emotional expressions, as facial movements and gestures often directly reflect a person’s emotional state (Table 4). Techniques such as facial landmark detection are commonly used to identify key points on the face (e.g., corners of the mouth, eyes, and eyebrows), which are then used to quantify facial expressions [24]. Histogram of Oriented Gradients (HOGs) is another widely used technique that captures the distribution of intensity gradients and edges in an image, providing a robust representation of facial features [50]. Deep learning approaches, especially CNNs, have been extensively used to automatically extract features from facial images, enabling models to learn complex patterns associated with different emotions [51]. Additionally, optical flow can be employed to capture motion patterns in facial expressions, enhancing the temporal understanding of emotional changes over time [52]. Beyond facial features, body posture and gestures can also be informative visual features, offering a more holistic view of a person’s emotional state [53].

**Table 4:** Summary of visual feature extraction methods.

Input Type	Type of Features	Feature Extraction	Publications
Facial images	Landmark points	Facial landmark detection	[24,54]
Facial images	Gradient features	HOG	[40,50,55]
Facial images	Deep features	CNN	[41,42,51]
Motion patterns	Temporal dynamics	Optical flow	[38,43,52]
Full body/skeleton images	Pose keypoints	Body-pose estimation (OpenPose, HRNet, etc.)	[53,56]
Gesture sequences	Gesture trajectories	Skeleton-based gesture recognition (ST-GCN, 2s-AGCN, etc.)	[57,58]
Eye region/gaze data	Gaze direction and eye movement	Video/IR eye-tracking, pupil detection	[18,59]

Text Feature Extraction: textual features are valuable for capturing the emotional content conveyed through spoken or written language (Table 5). Traditional text features include Bag-of-Words (BoWs) and Term Frequency–Inverse Document Frequency (TF-IDF) representations, which encode text as numerical vectors and allow further analysis [49]. More advanced methods involve word embeddings (e.g., Word2Vec, GloVe) that capture semantic relationships between words, thereby detecting subtle emotional cues in text [60]. Sequence models such as RNNs, particularly LSTMs, have been used to capture contextual dependencies in text, improving emotion detection from dialogue or written content [61]. Transformer-based models such as BERT have also been applied to extract contextual and sentiment features from text, achieving state-of-the-art performance in emotion recognition tasks [49]. Additionally, using sentiment lexicons—predefined dictionaries of words rated by emotional sentiment—can enhance text-based emotion recognition by incorporating external knowledge of word affect [62].

**Table 5:** Summary of text feature extraction methods.

Type of Features	Feature Extraction	Publications
Lexical features	Bag-of-words, TF-IDF	[39]
Semantic features	Word embeddings (Word2Vec, GloVe)	[44]
Contextual features	RNN, LSTM	[28,61,63]
Contextual sentiment	Transformer (BERT), sentiment lexicons	[45,46,49,62]
Self-supervised contextual	Fine-tuning large language models (RoBERTa, XLNet, GPT)	[64,65]
Prompt-based adaptation	Prompt learning/in-context tuning	[53,66]

In MER, combining features from different modalities is essential to capture the full spectrum of emotional expression. Feature level fusion involves concatenating features from different modalities before classification, and it has been shown to improve recognition accuracy in many cases [40]. Additionally, deep multimodal learning approaches learn joint representations of features from speech, visual, and text data, enabling models to capture cross-modal interactions effectively [67]. Techniques such as attention mechanisms have also been applied at the feature level to weigh the importance of features from each modality, leading to more accurate emotion recognition by focusing on the most relevant signals [5].

### 3.5. Additional Modalities and Their Sensing Principles

Beyond the core channels of speech, vision, text, and EEG already summarized in Section 3.4, four further modality groups have become increasingly relevant to multimodal emotion recognition (MER) research. Each group is outlined below with its sensing principle, merit and key limitation.

Gaze/Eye-Tracking: infrared or RGB cameras estimate gaze direction, fixation length, blink rate, and pupil diameter. These metrics index attention allocation and sympathetic arousal; when fused with facial or EEG cues, they raise recognition accuracy, particularly for subtle or masked emotions [18]. Advantage: non-contact, millisecond-level reactions. Limitation: needs unobstructed eyes and recalibration; gaze patterns vary widely across tasks and cultures.

Environmental Context: scene cameras and ambient microphones quantify light, crowd density, noise, or music genre; metadata (time, location) adds further situational cues. Context features help disambiguate similar expressions [68]—for example, a smile in a funeral scene versus at a party—improving robustness under “in-the-wild” conditions [69]. Advantage: supplies prior humans naturally used. Limitation: context sensing increases privacy risk, and errors propagate if the scene is misclassified.

Haptic/Tactile and Peripheral Physiology: Wearable or seat-embedded pressure sensors capture force, tremor, and posture changes; galvanic skin response, heart rate variability, or skin temperature reflect autonomic arousal. Such signals are difficult to fake and have proven complementary to audio-visual streams in edge-deployed MER systems [70]. Advantage: objective, user-independent arousal markers. Limitation: mostly encode intensity, not valence; require on-body hardware and careful artifact removal (motion, temperature).

Emerging Sensors:

Wearable multi-biosensors: smartwatches combine photoplethysmography, accelerometers, and temperature for continuous affect logging [21].

fNIRS headbands: near infrared light tracks frontal cortex blood oxygenation linked to affective appraisal [71].

Doppler and mm-wave radar: contactless measurement of micro-vibrations from respiration and heartbeat enables covert emotion screening, useful where cameras are unsuitable [72].

Advantages: operate in everyday settings, add neural or physiological depth, and can work when faces are hidden. Limitations: many are still experimental, sensitive to motion or multipath interference, and dependent on user acceptance of wearables.

### 3.6. An Integrative Taxonomy of the MER Landscape

To help readers navigate the rapidly expanding literature, we propose a three-dimensional taxonomy that organizes multimodal emotion recognition research by (i) sensed modality, (ii) computational method, and (iii) application context. Along the modality axis, work ranges from “conventional” audio-visual inputs to physiological channels (EEG, ECG, EDA) and recently to gaze, haptic cues, ambient context, and emerging contactless sensors such as millimeter-wave radar [12,21]. The methodological axis reflects a progression from early concatenation or statistical fusion, through late decision aggregation, to intermediate representations that exploit attention, graph contrastive learning, or cross-modal transformers. Channel-attention networks dominate physiological fusion—demonstrated by SCA-Net++ on SEED-IV [73]—whereas cross-modal transformers achieve the current in the wild state-of-the-art on ABAW and MELD benchmarks [16,74]. A growing branch of lightweight CNN-LSTM hybrids targets real-time edge devices, trading some accuracy for latency and energy gains [12].

The application axis spans offline corpus analysis, real-time human–computer interaction, emotion-aware robotics, and pervasive health monitoring. Dialogue centric systems (e.g., JOYFUL on MELD) emphasize trimodal text audio vision pipelines for social AI [74,75], while social-robot platforms such as Pepper integrate vision, speech, and context to adapt robot behavior in public spaces [15]. Wearable biosensor ensembles have opened continuous affect tracking for stress management and telehealth scenarios, outperforming camera-based solutions in privacy-sensitive contexts [70]. Mapping the literature onto this three-way grid reveals clear trends: the richest modality combinations increasingly couple with transformer or graph-based alignment, and these, in turn, enable deployment in unconstrained, high-impact settings such as open world video diaries [76] or human–chatbot field studies [77]. The taxonomy therefore not only summarizes the field but also highlights the converging trajectory toward deeply fused, context-aware, and application-specific MER systems.

## 4. Multimodal Information Fusion for MER

### 4.1. Bimodal Emotion Recognition

Bimodal emotion recognition involves combining two different modalities—such as speech and visual data—to improve the accuracy and robustness of emotion recognition systems (Table 6). By integrating features from two sources, bimodal approaches capture complementary information that enhances the system’s ability to recognize emotions more effectively than unimodal systems [25].

A common bimodal pairing is speech-visual emotion recognition, which combines facial expressions with speech signals to infer a person’s emotional state [26]. Facial expressions provide direct visual cues about emotions, while speech features (tone, pitch, prosody) convey emotional context through voice. Using both modalities together yields a richer representation of emotion, especially in cases where either modality alone may be ambiguous or insufficient [78].

Fusion strategies for bimodal MER generally fall into three categories: early fusion, late fusion, and hybrid fusion. In early fusion, features from both modalities are concatenated and fed into a classifier together, capturing feature level interactions between modalities. This approach can create a more holistic representation of emotion, but it may face challenges due to different feature dimensions and the need for precise synchronization of data [53]. In late fusion, separate classifiers are trained on each modality, and their outputs (e.g., predicted emotion probabilities) are combined via averaging or a meta-classifier to make the final decision [79]. For instance, Wear-BioNet fuses the soft probabilities of heart rate, electrodermal activity, and accelerometer CNN–GRU branches by simple arithmetic mean and attains 84.5% accuracy on WESAD [70], whereas EAR-RoBERTa first produces text specific and metadata-specific probability vectors and then feeds them to a logistic regression meta-classifier that pushes CMU-MOSEI accuracy to 81.9% [65]. Late fusion is flexible, allowing each modality to be processed independently, but it might miss nuanced cross-modal interactions. Hybrid fusion attempts to leverage the advantages of both early and late fusion by combining information at multiple stages; for example, using feature fusion followed by an additional fusion of intermediate or output representations. When properly implemented, hybrid fusion can exploit the complementary characteristics of each modality’s information [36].

Deep learning techniques have significantly advanced bimodal emotion recognition. CNNs are commonly used for extracting visual features, while RNNs or LSTMs capture temporal dependencies in speech data [38]. Attention mechanisms have also been introduced to assign different weights to each modality, enabling the system to emphasize the most informative features for emotion recognition [74]. This has improved the interpretability of bimodal systems by highlighting which features (facial cues or vocal intonations) contribute most to the emotion prediction. For example, an attention-based model might focus on facial features when visual emotion cues are strong and rely more on vocal tone when the face is less expressive.

Another strategy in bimodal MER is the use of transfer learning between modalities. For instance, a model pre-trained on a large facial expression dataset might be fine-tuned on an audio emotion dataset (or vice versa), effectively transferring knowledge from one modality to improve performance on the other [72]. One study pre-trained a speech encoder on 650 h of VoxCeleb and, after freezing the lower layers, fine-tuned it jointly with a visual stream, improving F1 on CREMA-D from 73.2% to 77.5% [36]. A second work (MemoCMT) initializes its video backbone from ImageNet and then adapts it together with audio and text specific transformers, reaching 82.3% accuracy on CMU-MOSEI [58]. Such transfer learning approaches leverage existing learned representations and can be particularly useful when one modality has limited labeled data.

Cross-modal learning is another important concept, where features of one modality are used to inform feature extraction in another modality. For example, visual features could guide the extraction of relevant speech features, resulting in more accurate emotion recognition [80]. By bridging the gap between modalities, cross-modal learning improves the system’s robustness, ensuring that information from one modality can compensate for weaknesses in the other.

Bimodal fusion techniques also include methods such as canonical correlation analysis (CCA), which finds linear correlations between feature sets of different modalities to create a shared representation space for both [53]. CCA-based methods increase recognition capability by focusing on information common to both modalities, making it easier to detect emotions that manifest in correlated audio-visual patterns.

**Table 6:** Summary of bimodal emotion recognition methods.

Input Modality 1	Input Modality 2	Output Emotion	Fusion Strategy	Methods Used	References
Speech	Visual	Discrete emotions	Early fusion	CNN for visual, LSTM for speech	[12,25,34]
Speech	Visual	Continuous emotions	Late fusion	RNN for speech, SVM for visual	[28,52,65,70,79]
Visual	Physiological	Discrete emotions	Hybrid fusion	HOG for visual, CNN for signals	[42,53]
Speech	EEG	Arousal and valence	Early fusion	Spectrogram analysis, CNN	[48,74]
Text	Visual	Positive/negative	Attention mechanism	Transformer, attention network	[10,36,58]
Speech	Visual	Discrete emotions	Transfer learning	Pre-trained ResNet, RNN	[36,38,72,81]
Audio	Visual	Emotional state	Cross-modal Learning	CCA, CNN	[52,80]
Visual	Physiological	Stress detection	Hybrid fusion	LSTM for temporal, SVM	[40,82]
Speech	Visual	Empathy detection	Late fusion	CNN, random forest classifier	[26,28]
Text	Facial expressions	Sentiment analysis	Early fusion	TF-IDF for text, HOG for visual	[48,67]

### 4.2. Trimodal Emotion Recognition

Trimodal emotion recognition involves the integration of three different modalities—such as speech, visual, and text data—to provide a more holistic view of human emotions (Table 7). By combining three modalities, trimodal systems capture diverse aspects of emotional expressions from multiple sources. This approach can compensate for the limitations of individual modalities, leading to improved accuracy and robustness in emotion recognition systems.

One of the most common trimodal combinations is speech, visual, and text. Using these methods together works well for discovering emotions in programs where people interact, because they include voice quality, facial expressions, and spoken content. Facial expressions and delineated parts of the face give us direct advice on emotions, pitch and energy in speech offer sound style, and text provides context through meaning [61]. Using them together helps us discover and explore emotions, especially when we deal with multiple peers [79]. This rationale directly aligns with the public corpora reviewed in Section 3.3. GEMEP couples high-resolution facial action units with synchronized speech, making it ideal for probability averaging of visual and acoustic streams [41]. K-EmoCon augments those channels with wearable biosignals, enabling late fusion meta-classifiers that exploit arousal information unavailable in the face or voice alone [42]. For dialogue-centric tasks, IEMOCAP and MELD add textual transcripts so that lexical context can refine ambiguous prosodic cues [43,45].

Typical approaches to trimodal fusion are feature level fusion, decision-level fusion, and hybrid fusion. Features from each of the three modalities are joined together to represent a single feature vector for classifying emotions. When using this approach, the model is able to learn about all the data sources at once; however, because the data is very large, it must be correctly aligned and normalized [83]. Alternatively, decision-level fusion teaches each modality to make its own predictions and merges those results together, for example, using voting or weighted calculations. With this strategy, various data types are easier to work with, but it may not catch all the mixed-modality relationships as well as the other approach does [84]. Hybrid fusion uses both types of fusion and combines information in several places as the data flows through the system. Example approaches often start by joining the information from two forms of data and end by merging that intermediate result with data from the third source in the decision stage [67]. This method is designed to connect the unique advantages found in each fusion process [39].

Deep learning has been widely utilized in trimodal MER. For example, CNNs can extract visual features, while RNNs or transformer models capture temporal and contextual information from speech and text, respectively [80]. Attention mechanisms in trimodal systems can assign different importance weights to each modality, enabling the model to concentrate on the most relevant features for emotion recognition [74]. This helps in effectively handling cases where one modality might be noisy or less informative. For instance, if background noise affects the audio quality, the model can rely more on visual and textual cues [85].

Another technique in trimodal MER is modality alignment, where features from different modalities are projected into a common feature space to simplify fusion. Techniques such as dimensionality reduction (e.g., canonical correlation analysis, CCA) can be applied to aligned features, reducing complexity and ensuring that shared information across modalities is emphasized [53]. Aligning the features can enhance the relationships captured among modalities, leading to better emotion recognition performance [43].

Cross-modal transformers have recently gained popularity in trimodal MER, as they are capable of modeling complex relationships among multiple modalities. These models use self-attention mechanisms to learn interactions between speech, visual, and text features, thereby capturing the intricate dynamics of human emotions [79]. Cross-modal transformers have shown promising state-of-the-art results on emotion recognition benchmarks, demonstrating their ability to handle the rich and dynamic nature of multimodal emotional data [32].

Table 8 compares recent state-of-the-art MER systems across datasets, fusion strategies and metrics, illustrating that transformer-based cross-modal models consistently outperform conventional early fusion on large-scale benchmarks.

Table 9 summarizes, across major modality classes, the state-of-the-art aggregation methods and their best reported performance, highlighting that channel-attention mechanisms dominate physiological fusion, whereas graph contrastive or transformer architectures excel in trimodal dialogue settings.

**Table 7:** Summary of trimodal emotion recognition techniques.

Modality 1	Modality 2	Modality 3	Fusion Strategy	Methods Used	References
Speech	Visual	Text	Decision-level	SVM/random forest + RNN	[51,61]
Speech	Visual	Text	Early-level (feature concat.)	CNN (faces) + Log-Mel spectrogram	[83]
Speech	Visual	Text	Cross-modal transformer	CCA pre-alignment + transformer	[84,86]
Audio	Visual	Text	Attention mechanism	CNN + cross-modal attention	[85,87]
Text	Speech	Visual	Cross-modal transformer	Self-attention encoder	[74,84]
Visual	Physiological	Text	Hybrid (feature + decision)	HOG + LSTM + sentiment lexicon	[53,79]
Speech	Visual	Physiological	Hybrid (ResNet → LSTM)	Pre-trained ResNet (face) + LSTM (biosignal)	[61,88]
Speech	EEG	Visual	Modality alignment	CCA + CNN	[80,89]
Visual	Text	EEG	Feature-level	CNN (image) + BERT (text) + spectrogram	[60,79]
Text	Facial expressions	Speech	Decision-level	TF-IDF + CNN + GRU	[52,90]
Speech	Visual	Text	Ensemble hybrid (graph contrastive + transformer)	JOYFUL/graph contrastive alignment	[39]
Speech	Visual	Text	Cross-modal transformer (state-of-the-art)	Self-attention fusion	[32,43]

**Table 8:** Comparative table of performance metrics with aggregation methods.

Study	Modalities	Public Dataset	Aggregation/Fusion Method	Metric (Type)	Reported Score
TMNet [20] (2025)	Speech + EEG	SEED-IV	Cross-modal transformer (early + attention)	Acc.	88.70%
MemoCMT [58] (2025)	Vision + speech + text	CMU-MOSEI	Cross-modal transformer	Acc.	82.30%
JOYFUL [74] (2023)	Audio + text + vision	MELD	Graph contrastive mid-level fusion	F1-macro	81.20%
Edge-MER [12] (2024)	Facial + audio	RAVDESS	Lightweight CNN-LSTM (early)	WA	79.40%
EAR-RoBERTa [65] (2023)	Text (+ meta)	CMU-MOSEI	Emotion-specific attention (late)	Acc.	81.90%
Joint-MMT [16] (2024)	Vision + speech + action	ABAW 2023	Unified transformer (late)	F1-macro	48.90%
Interp-Hybrid [72] (2021)	Vision + speech + text	IEMOCAP	Hybrid early-/late + heat-map attention	Acc.	82.00%
FG-Disentangle [91] (2022)	Audio + text + vision	MELD	Disentangled representation (early)	F1-macro	80.10%
Wear-BioNet [70] (2025)	Wearable HR + EDA + accel.	WESAD	Ensemble CNN-GRU (late)	Acc.	84.50%

**Table 9:** Modality–method–performance Matrix.

Modality (Primary Channels)	Typical Evaluation Setting	Method Class with Best-Reported Result	Representative Study [Dataset]	Key Metric	Best Score
Audio + visual	In-the-wild video (ABAW 24)	Cross-modal 3-D transformer fusion	Cross-modal 3D Facial-Speech [72]	F1-macro	0.83
Audio + visual + text	Multi-speaker dialogue (MELD)	Graph contrastive mid-level fusion	JOYFUL (MELD) [74]	F1-macro	0.812
Physiological (EEG/facial)	Lab elicitation (SEED-IV)	Channel-attention early fusion	SCA-Net++ [92]	Accuracy	0.912
Wearable biosensors (HR, EDA, Accel.)	Real-life stress (WESAD)	Ensemble CNN-GRU, late fusion	Wear-BioNet [70]	Accuracy	0.845
Gaze + audio + text	Human‚ Äìchatbot field study	Hybrid attention, early + late	Multimodal Cues Chatbot [77]	F1	0.74
Edge-device AV	Resource-limited real-time	Lightweight CNN-LSTM, early	Edge-MER (RAVDESS) [12]	WA	0.794

## 5. Research Challenges and Open Issues

### 5.1. Lightweight and Explainable Deep Models for MER

The development of lightweight and explainable deep models is crucial for making MER systems more accessible and interpretable. Model interpretability is especially important in sensitive fields such as healthcare and education, where understanding how the model arrives at decisions can foster trust and improve user acceptance [5]. One major challenge in MER is the high computational cost of deep learning models, which often limits their deployment on edge devices with limited resources. Lightweight models (e.g., using pruning, quantization, or knowledge distillation techniques) have been explored to reduce computational burden without significantly compromising accuracy [93].

In parallel, explainable artificial intelligence (XAI) methods (such as attention visualization, saliency maps, or feature attribution techniques) have been employed to make MER models more transparent, allowing users to understand which features or modalities are most influential in the emotion recognition process [44]. Combining lightweight architectures with explainability is a promising direction to make MER systems user-friendly and viable for real-world scenarios [94].

### 5.2. Multimodal Information Fusion Strategies

While multimodal information fusion is key to MER, it presents challenges such as differing temporal resolutions, noise levels, and missing data across modalities. Early fusion (feature concatenation) can struggle with the high dimensionality of combined features, making model training difficult [95]. Late fusion (combining classifier outputs) might lose important cross-modal interactions that are essential for recognizing complex emotional cues [33]. To address these challenges, hybrid fusion techniques have emerged, aiming to leverage the strengths of both early and late fusion by combining information at multiple stages. Hybrid fusion enables models to capture both intra- and inter-modal relationships effectively [46]. To capture deeper inter-dependences, Praveen and Alam devised a Recursive Joint Cross-Modal Attention mechanism that iteratively refines intra- and inter-modal correlations and sets new CCC records on dimensional emotion tasks [96].

Recent advances in deep learning, such as attention-based fusion methods, have shown significant promise in multimodal fusion by assigning different weights to each modality based on its relevance to the emotion being recognized [97]. Additionally, transformer architectures have been used to model complex dependencies among modalities, allowing for more flexible and powerful fusion mechanisms [74]. These approaches help overcome limitations of traditional fusion by dynamically focusing on the most informative signals. Another approach involves using cross-modal transformers, which can capture both individual modality features and their interactions, providing a comprehensive representation of emotions [69].

### 5.3. Cross-Corpus MER

A major challenge in MER is the lack of generalizability across different datasets, often referred to as the cross-corpus problem. Models trained on one dataset often perform poorly on others due to variations in recording conditions, cultural differences, and emotional expression styles [98]. This highlights the need for MER systems that generalize well across different contexts and populations. Transfer learning and domain adaptation techniques have been explored to tackle this problem by enabling models to leverage knowledge from one dataset to improve performance on another [40].

Adversarial training targets dealing with the gap between the features of the source and the target dataset in order to improve cross-corpus generalizability. The model is less affected by biases in a dataset when it can identify domain-invariant features [99]. Moreover, using synthetic data or style transfer boosts the variety of the training set, which helps models withstand differences in real-world data [67]. The AVaTER framework fuses audio, visual and textual cues through a compact cross-modal attention block, achieving real-time inference on edge GPUs while improving accuracy across four public corpora [100,101].

### 5.4. More Modalities for MER

Incorporating additional modalities (e.g., physiological signals or contextual data) can enhance the accuracy and robustness of MER systems by providing richer information about the user’s emotional state. Signals from the heart, skin, and brain all show how the autonomic nervous system is involved in controlling emotions [100]. Supplementing signals from wearables with speech and video helps MER systems better understand how people feel.

Environmental factors and what is happening around us can provide valuable information too. Identifying emotions in a loud and jam-packed environment might need other procedures than it does in a calm, quiet location [102]. When you supply the environment, time, and presence of others, the machine can more accurately detect your moods. Thanks to recent progress in sensors, MER systems can now monitor and react to a person’s state and the environment in real-time [32].

### 5.5. Few-Shot Learning for MER

A significant challenge in MER is the scarcity of labeled data, which limits the training of deep learning models. Few-shot learning (FSL) is an emerging approach that addresses this by enabling models to learn from only a handful of labeled examples. FSL methods (such as metric learning or prototypical networks) have been applied to MER to recognize emotions with minimal data [19]. By learning a feature space where examples of the same emotion are clustered together, these models can generalize to new emotion classes with only a few examples [47].

Another promising approach is the use of generative models (such as variational autoencoders or generative adversarial networks) to create synthetic training data, augmenting the limited real data [50]. This can mitigate data scarcity by providing additional samples for training, thereby improving model robustness. Additionally, transfer learning from related domains (e.g., using models pre-trained on sentiment analysis or facial recognition tasks) can provide a strong initialization for MER models, which can then be fine-tuned on small emotion datasets [83].

Self-supervised learning is another avenue, where models learn to extract meaningful features from unlabeled data through proxy tasks (such as reconstructing missing parts of input or predicting temporal order). Such methods have shown promise in MER by leveraging large amounts of unlabeled multimodal data to pre-train representations that are later fine-tuned on the small, labeled emotion datasets [103]. Collectively, these approaches make MER systems more feasible in scenarios where labeled data is scarce, expanding their applicability to new domains and populations.

## 6. Summary

This review has highlighted key advancements and challenges in multimodal emotion recognition (MER). Integrating multiple modalities—such as speech, visual, and text—has proven to be an effective strategy for capturing the complexity of human emotions and improving recognition accuracy. Bimodal and trimodal approaches demonstrate significant improvements over unimodal systems by leveraging complementary information from different data sources, thus providing a more comprehensive understanding of emotions [8]. The use of deep learning techniques (CNNs, RNNs, and transformers) has further enhanced MER performance by enabling the extraction of complex features and modeling interdependencies among modalities [61].

Multimodal information fusion remains a critical aspect of MER, with various fusion strategies (early, late, and hybrid) employed to combine information from different modalities. Hybrid fusion has shown promise in capturing both intra-and inter-modal relationships, thereby improving the robustness of MER systems [40]. Recent advances, such as attention-based fusion and cross-modal transformers, have further enhanced multimodal fusion effectiveness by allowing models to focus on the most relevant features and capture complex dependencies among modalities [32].

Despite the progress, several challenges remain in MER. Developing lightweight and explainable deep models is essential for making these systems more accessible and trustworthy, especially in sensitive applications such as healthcare and education [19]. The high computational cost of deep models poses a barrier to deployment on edge devices, necessitating efficient, lightweight architectures [86]. Additionally, the lack of interpretability in many deep learning models hinders their acceptance, highlighting the need for XAI techniques to provide insights into the decision-making process [93].

Cross-corpus generalizability is another major challenge: models often struggle on datasets other than the ones they were trained on due to differences in conditions and expression styles [94]. Techniques such as transfer learning, domain adaptation, and adversarial training have been proposed to improve MER robustness across contexts [95]. Moreover, incorporating additional modalities such as physiological and contextual data can enrich MER systems by providing deeper insight into users’ emotional states, making the systems more adaptable to different scenarios [32].

Future research in MER should focus on addressing these challenges by developing more efficient, explainable, and generalizable models. The integration of non-traditional modalities (e.g., physiological signals, context) holds great potential for enhancing accuracy and robustness. Furthermore, few-shot and self-supervised learning techniques offer promising solutions for the data scarcity problem, potentially broadening the applicability of MER to new domains and populations [34]. Practical implementations of MER should also consider user-centric design, ensuring that models are not only accurate but also interpretable and accessible to end users. By meeting these challenges and leveraging recent advancements in deep learning and sensor technology, MER has the potential to play a significant role in enhancing human–computer interaction, healthcare, education, and other fields where understanding human emotions is crucial.

## Data Availability

Data sharing is not applicable.
